# Composition, Antibacterial Efficacy, and Anticancer Activity of Essential Oil Extracted from *Psidium guajava* (L.) Leaves

**DOI:** 10.3390/plants12020246

**Published:** 2023-01-05

**Authors:** Aftab Alam, Talha Jawaid, Saud M. Alsanad, Mehnaz Kamal, Mohamed F. Balaha

**Affiliations:** 1 Department of Pharmacognosy, College of Pharmacy, Prince Sattam Bin Abdulaziz University, Al-Kharj 11942, Saudi Arabia; 2 Department of Pharmacology, College of Medicine, Imam Mohammad Ibn Saud Islamic University (IMSIU), Riyadh 13317, Saudi Arabia; 3 Department of Pharmaceutical Chemistry, College of Pharmacy, Prince Sattam Bin Abdulaziz University, Al-Kharj 11942, Saudi Arabia; 4 Department of Clinical Pharmacy, College of Pharmacy, Prince Sattam Bin Abdulaziz University, Al-Kharj 11942, Saudi Arabia; 5 Pharmacology Department, Faculty of Medicine, Tanta University, Tanta 31527, Egypt

**Keywords:** *Psidium guajava*, essential oil, leaves, oral infection, molecular coupling, cytotoxic effects

## Abstract

Essential oils (EO) are used as a natural remedy to treat various chronic diseases, although clinical evidence is lacking. In this context, we have endeavored to measure the percentage of chemical composition and biological efficacy of *Psidium guajava* (guava) leaf essential oil in treating oral infections and oral cancer. The essential oil obtained from hydrodistillation of *P. guajava* L. leaves was analyzed by gas chromatography–mass spectrometry (GC–MS). The activities of selected oral pathogens *Candida albicans* (*C. albicans*) and *Streptococcus mutans* (*S. mutants*) were studied in vitro and in silico. MTT assay was used to test for anticancer activity against human oral epidermal carcinoma (KB). GC–MS showed that the main components of PGLEO were limonene (38.01%) and β-caryophyllene (27.98%). Minimum inhibitory concentrations (MICs) of 0.05–0.1% were demonstrated against *C. albicans* and *S. mutans*. Antimicrobial activity against *C. albicans* and *S. mutans*, as shown by molecular linkage analysis, revealed that the main metabolites, limonene and β-caryophyllene, potentially inhibited the receptors of *C. albicans* and *S. mutans*. PGLEO showed significant (*p* < 0.001) anticancer activity (45.89%) at 200 µg/mL compared to doxorubicin (47.87%) with an IC_50_ value of 188.98 µg/mL. The outcomes of the present study suggest that PGLEO has promising antimicrobial and anticancer activities and could be a useful source for developing a natural therapeutic agent for oral infections and oral cancer.

## 1. Introduction

*Psidium guajava* L. (*P. guajava*), a tropical and subtropical plant of the Myrtaceae family, is commonly called guava and is used for nutritional and medicinal purposes. In traditional medicine, the fruits, leaves, and juice of guava are commonly used to treat oral and dental infections, pain, and other diseases [1]. The leaves of this plant are traditionally used in the African and Asian continents to treat various infectious diseases [2]. The aqueous leaf extract is effective against various Gram-positive/Gram-negative bacteria, fungi, viruses, and protozoa [3].

The use of *P. guajava* is found in various traditional systems of medicine, including the Ayurvedic system of medicine of India, the traditional Chinese system of medicine, and the folk systems of medicine of the West Indies and Latin America. Guava leaves have been studied for various activities, including antioxidant, antidiabetic, antihyperlipidemic, antidiarrheal, cardioprotective, analgesic, nephrotoxic, and antimicrobial effects [4]. Toxicity studies have shown that *P. guajava* is safe to use [5]. The leaf extract of guava has been reported for its anticancer and antibacterial activities [6]. Recently, several terpenoid compounds have been isolated from the leaf extract of guava and evaluated. Among the isolated compounds, several meroterpenoids showed anticancer and antifungal activities [7,8]. The essential oil constituents depend on the chemical composition, geographical conditions, growing conditions, and collection time of the mother plant and its parts [9,10].

Guava leaves from different regions have different volatile compositions; for example, Chinese guava contains β-caryophyllene, copaen, azulene, eucalyptol, etc. [11]; Brazilian guava contains β-caryophyllene, β-elemene, β-selinol, and α-humine [12]; and Egyptian guava contains β-caryophyllene, trans-nerolidol, globulol, and D-limonene [13]. Recently, Indian guava was shown to contain limonene, caryophyllene, caryophyllene oxide, etc. [14]. In general, terpenoids, such as limonene, β-caryophyllene, 1,8-cineole, β-elemene, etc., may be responsible for antimicrobial and anticancer activities [15,16].

The extensive use of conventional antimicrobials in oral infections has led to antimicrobial resistance. The rate of antimicrobial resistance is higher than that of new drug development, which poses a medical challenge [17,18]. Guava leaves are commonly used to treat mouth ulcers in southern India, while in northern India, raw leaves and tender shoots of guava are used to treat toothache and mouth ulcers [18]. Traditional Cameroonians use guava leaves to treat dental infections, and guava twigs are chewed to relieve dental problems [19]. Extracts of guava leaves with various solvents have shown antibacterial activity against oral pathogens and dental plaque [20]. Various microorganisms are present in the oral cavity; *Streptococcus mutans* and *Candida albicans* play a significant role in developing oral cavity, caries, and periodontal infection [21]. Evaluating molecular linkage studies is an effective tool for drug design and development [20,21,22]. Human epithelial carcinoma cells (KB cell lines) are squamous cell carcinoma (SCC). They are the most common oral cavity cancer responsible for morbidity and mortality [23]. In a previous study, essential oil from 17 Thai medicinal plants was investigated against KB cell lines, including oil from guava leaves. It was reported that oil from guava leaves had the highest antiproliferative values, more than sweet basil and vincristine [24].

Considering the enormous amount of research on the antimicrobial and anticancer effects of guava essential oil, this study aimed to investigate the role of PGLEO in protecting against oral infections and oral cancer. In addition, molecular linkage studies against *S. mutans* and *C. albicans* receptors were performed to validate the mechanism of antimicrobial activity of the main compounds analyzed in PGLEO. In addition, the anticancer effect on human oral epidermal carcinoma cell lines (KB) was investigated using an MTT assay to determine the role of PGLEO in protecting against oral cancer and bacterial infections. 

## 2. Results

### 2.1. Characterization of Essential Oil Composition through Gas Chromatography–Mass Spectrometry (GC–MS)

**Figure 1:**
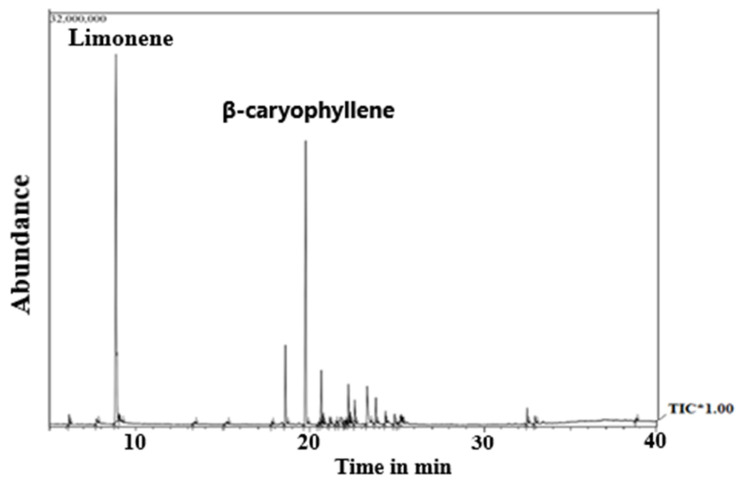
GC–MS chromatogram of PGLEO.

The *Psidium guajava* leaves produced 0.58% *w*/*w* of essential oil. Figure 1 shows the gas chromatography–mass spectrometry chromatogram of PGLEO. A total of 34 volatile metabolites were identified and are listed in Table 1. Among the volatile essential oils, the total concentration of monoterpenes was 40.37%, of which monoterpene hydrocarbons and oxygenated monoterpenes accounted for about 39.87% and 0.5%, respectively.

The *Psidium guajava* leaves produced 0.58% *w*/*w* of essential oil. Figure 1 shows the gas chromatography–mass spectrometry chromatogram of PGLEO. A total of 34 volatile metabolites were identified and are listed in Table 1. Among the volatile essential oils, the total concentration of monoterpenes was 40.37%, of which monoterpene hydrocarbons and oxygenated monoterpenes accounted for about 39.87% and 0.5%, respectively.

However, the total concentration of sesquiterpenes was 57.65%, of which about 49.54% were sesquiterpene hydrocarbons and 8.11% were oxygenated sesquiterpenes. Six main volatile constituents (<3%) were detected in the present sample, including five sesquiterpenes (β-caryophyllene (27.98%), copaene (6.25%), cubenene (4.80%), nerolidol (4.49%) and α-Caryophyllene (4.21%)) and one monoterpene (D-limonene (38.01%)). These compounds accounted for about 85.81% of the total percentage of volatile compounds.

### 2.2. Antibacterial and Antifungal Activities against Oral Bacteria

The results of PGLEO antimicrobial assays against *S. mutans* and *C. albicans* are summarized in Table 2. An inhibition zone of 6 mm in diameter was considered a positive result. The zone of inhibition study outcomes showed that PGLEO exhibited significant inhibitory activity against *S. mutans* (18.5 ± 0.7 mm) and *C. albicans* (21.5 ± 0.5 mm) at 10%. The outcomes of minimum inhibitory concentration (MIC: 0.05–0.1%) showed that PGLEO exhibited almost equal activity against both *S. mutans* and *C. albicans*.

**Figure 2:**
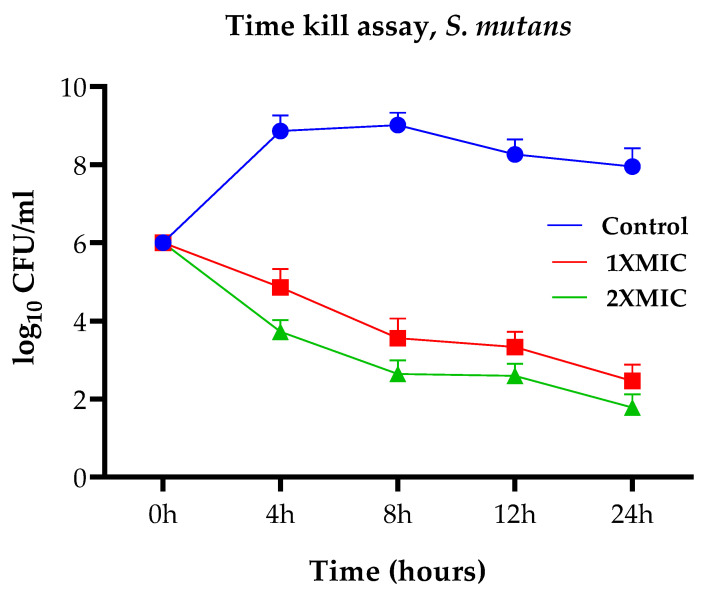
Time-kill assays of *S. mutans*.

**Figure 3:**
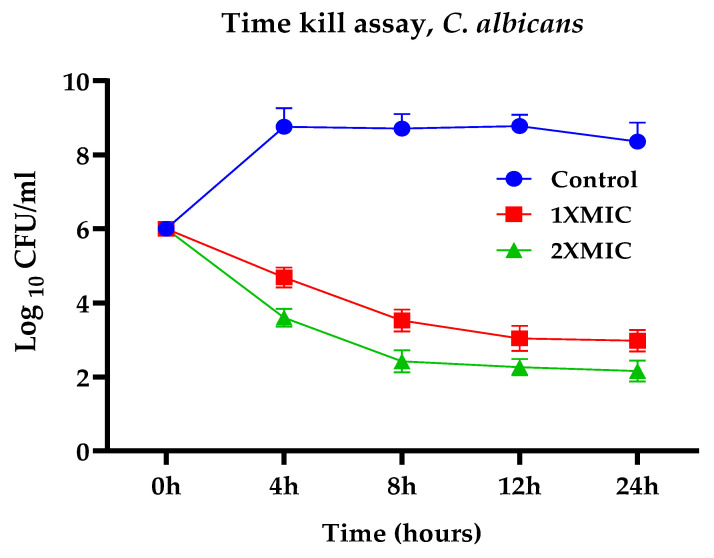
Time-kill assays of *C. albicans*.

The cell viability (time-kill) assays were performed using PGLEO, and the results are shown in Figure 2 and Figure 3. The outcomes of the control group (untreated *S. mutans*) exhibited approximately 6 to 9 (log10 CFU/mL) growth. In contrast, the test group (PGLEO treated) showed that the growth of *S. mutans* decreased dramatically in the first 4 to 8 h. It remained constant at approximately 2.5 to 3.5 (log10 CFU/mL) and 1.8 to 2.6 (log10 CFU/mL) with 1XMIC (0.1%) and 2XMIC (0.2%) treatment, respectively (Figure 2). Similarly, untreated *C. albicans* grew at 6 to 8.7 (log10 CFU/mL). After treatment with PGLEO, the growth of *C. albican* decreased dramatically in the first 4 to 8 h. It remained constant at approximately 3 to 3.5 (log10 CFU/mL) and 2 to 2.4 (log10 CFU/mL) for 1XMIC (0.1%) and 2XMIC (0.2%) treatment, respectively (Figure 3). The results indicate that PGLEO exhibits a lethal effect on *S. mutans* and *C. albicans* equally. The plot of both samples measured at the 2XMIC stage differed from that at the 1XMIC stage for both microorganisms. PGLEO showed a rapid killing effect with 2XMIC.

### 2.3. Molecular Coupling Studies

Two important volatile compounds, D-limonene and β-caryophyllene, were selected for molecular coupling studies. Molecular coupling was performed against antioxidant target enzymes such as *S. mutans* antigen I/ II (PDB: 1JMM) and antifungal target enzymes such as N-myristoyl transferase receptors (PDB: 1IYL) to identify critical ligand–protein interactions. Table 3 shows the results of binding energy, inhibition constant, and amino acid residues.

**Figure 4:**
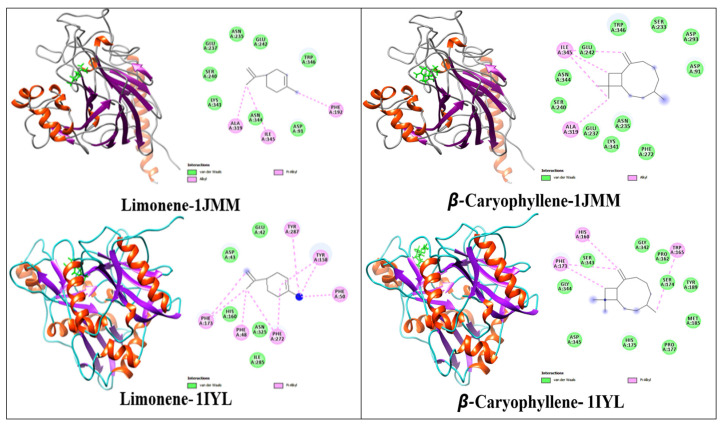
Interaction of the 1JMM and 1IYL protein with the ligand D-limonene and β-caryophyllene.

Figure 4 presents the ligand interaction of D-limonene (1-2) and β-caryophyllene (3-4) with proteins. The primary metabolite, limonene, displayed a high binding affinity to *Candida* receptors 1IYL with docking scores of 8.03, while the second major metabolite, β-caryophyllene, demonstrated higher docking scores of 8.21.

*S. mutans* receptor 1JMM and *Candida* receptor 1IYL showed a binding energy of 7.42 and −7.39 kcal/mol against limonene and β-caryophyllene, respectively. A similar trend in linkage values was observed for both limonene and β-caryophyllene. The Ki range of limonene and β-caryophyllene was highest against the Candida target 1IYL (640.45 µM) and moderate against the *S. mutans* target (752.10 µM). The Ki value against the *S. mutans* target (1JMM) was lower than against 1IYL.

### 2.4. Inhibition of KB Cells from the Growth of Oral Cancer by PGLEO

**Figure 5:**
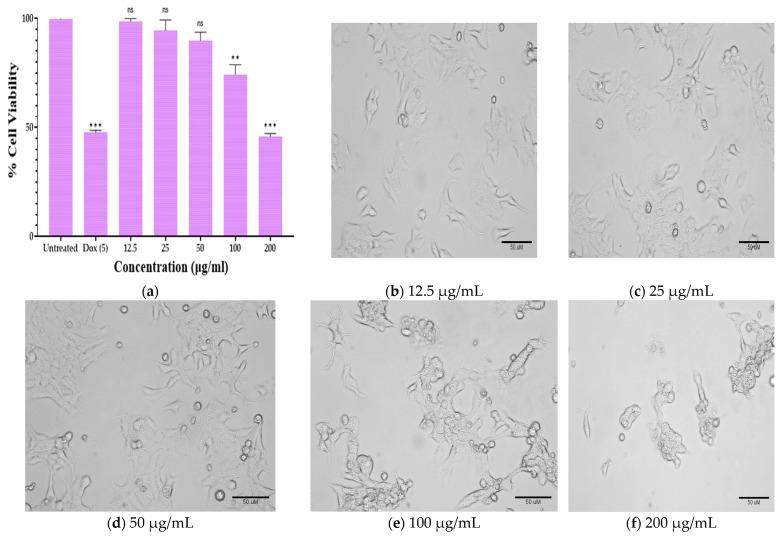
(**a**) Effect of PGLEO on cell viability and morphological characteristics; ** *p* < 0.05 and *** *p* < 0.001 compared to control. (**b**–**f**) KB cells treated with PGLEO at various concentrations (12.5 to 200 µg/mL) for 24 h.

Figure 5 shows the effects of PGLEO treatment at 25 to 200 µg/mL on cell viability measured by MTT assay from KB. PGLEO treatment significantly suppressed production of KB cells after 24 h of treatment (Figure 5A). In this study, the IC_50_ value of PGLEO was measured using a linear regression equation, i.e., Y = Mx + C. Here, Y = 50 and M and C values were derived from the viability graph. The survival of KB cells was significantly (*p* < 0.001) reduced in a dose-dependent manner with an IC_50_ value of 188.43 ± 8.26 µg/mL (Figure 5a). PGLEO treatment resulted in irregular morphology of KB cells, which shrank and exhibited a round shape, and reduced the viability of KB cells (Figure 5b–f).

The MTT assay is a colorimetric assay for determining cell proliferation and cytotoxicity based on reducing the yellow-colored water-soluble tetrazolium dye MTT to formazan crystals. Mitochondrial lactate dehydrogenase produced by living cells reduces MTT to insoluble formazan crystals. After dissolution in a suitable solvent, they show a purple color; the intensity is proportional to the number of viable cells and can be measured spectrophotometrically at 570 nm. The observations in the statistical data of the cell cytotoxicity study by MTT indicated that PGLEO had significant cytotoxic potential against KB cell lines and test compounds.

## 3. Discussion

The percentage yield of PGLEO was similar to a previous study with qualitatively different volatile compositions [25,26]. The major constituents of essential oil vary depending on the geographical location, cultivar, and cultivar of the parent plant. Several studies have been conducted on the essential oil of North Indian guava leaves. The results showed similar proportions of sesquiterpenes and monoterpenes but differences in the quality and quantity of constituents [26]. Soliman et al. (2016) reported a higher proportion of monoterpenes than sesquiterpenes; in contrast to the present study, sesquiterpenes were identified as the main constituent [27]. Has-san et al. studied the PGLEO of six cultivars from the same farms in Egypt. They showed that the Red Malaysian and White Indian cultivars contained limonene as the significant volatile compound. In contrast, El-Qanater, Early, El-Sabahya El-Gedida, and Red Indian had β-caryophyllene as the significant volatile compound [13].

Several studies have reported that caryophyllene is an essential component of PGLEO. Weli et al. studied indigenous PGLEO from the Sultanate of Oman (October 2012) and found that caryophyllene, viridiflorene, and farnesene were the major volatile compounds [28,29]. Chen and Yen (2007) studied the PGLEO of cultivars from Taiwan and found that β-caryophyllene, α-pinene, and 1,8-cineole were the major volatile compounds [30]. Satyal et al. (2015) investigated the essential oil composition of guava leaves from Kathmandu, Nepal. They reported that (E)-nerolidol and (E)-caryophyllene were the major compounds, which is in agreement with the results of the present study [31]. Silva et al. (2019) investigated the essential oil composition of guava leaves from Rio Verde Go, Brazil. They reported antibacterial activity against *S. mutans* that was similar to the present study, but the major volatile compositions of β-caryophyllene, α-humulene, aromadendrene oxide, δ-selinene, and selin-11-en-4α-ol were different [32].

Chalannavar et al. (2014) studied the essential oil composition of guava leaves from South Africa. They reported that sesquiterpenes, caryophyllene oxide, and caryophyllene were the main constituents, in contrast to the present study [33]. In addition, some studies have reported that limonene is the main constituent of PGLEO [34]. Chaturvedi et al. recently analyzed the composition of Indian guava essential oil. They reported that the quality and quantity of limonene, (E)-caryophyllene, and caryophyllene oxide were relatively different from those observed in the present study [14]. Sacchetti et al. studied the essential oil composition of guavas from the Philippines and found that limonene, α-pinene, β-caryophyllene, and longicyclene were the most significant compounds [35]. The highest contents of the monoterpenes limonene and α-pinene were found in PGLEO from Ecuador. In contrast, Tunisian guava leaf oil was found to have the highest content of the sesquiterpenes vi-ridiflorol and trans-caryophyllene [36]. Most studies have reported that caryophyllene is one of the main components of guava essential oil; the fragrance of guava leaves could be due to the combined effect of β-caryophyllene and other compounds [14,16,37].

Diseases found to have associations with the oral cavity, especially dental diseases but also diseases such as cardiovascular disease, diabetes, rheumatoid arthritis, pneumonia, and Alzheimer’s disease, are the main causes of high morbidity and mortality [38]. The oral cavity, especially saliva and dental plaque, of patients with periodontal disease or other oral infections seems to be a logical source of oral pathogens. Among the various microorganisms in the oral cavity, the caries bacteria *S. mutans* and the fungus *C. albicans* play an essential role in developing oral cavity and dental infections [39]. *S. mutans* is most commonly associated with dental caries [40]. Bhushan et al. (2014) reported the antidermatophytic activity of PGLEO against various fungal strains, suggesting that it has excellent antifungal potential [41]. Gonçalves et al. (2008) studied the antimicrobial activity of guava essential oils and reported that they have inhibitory activity against Gram-positive and Gram-negative bacteria [42]. Several studies have investigated the anticarcinogenic activity of PGLEO and reported the potential effects of Streptococcus sp. and Candida sp. against various oral pathogens and dental plaque, supporting the results of the present study [43,44]. The antimicrobial activities (*S. mutans* and *C. albicans*) of PGLEO may be attributed to a high percentage of significant compounds such as limonene, β-caryophyllene, or various other components [45,46,47,48].

Docking (molecular) studies have been widely used to evaluate the pharmacological properties of natural products; they explain the likely mechanisms of action and the binding pathways within protein pockets [49]. Previous studies have reported the role of *C. albicans* in dental plaque, caries lesions, and other types of oral infections [50,51,52]. Limonene and β-caryophyllene may have mechanisms against *C. albicans* N-myristoyl transferase) and *S. mutans* antigen III [53,54]. The molecular docking simulation results support the inhibitory activities of PGLEO against *S. mutans* and *C. albicans*. Previous in vitro antifungal and antibacterial studies have demonstrated the efficacy of PGLEO against oral infections [2,11,14,25]. The activity of PGLEO may be due to the presence of the significant compounds limonene and β-caryophyllene, which strongly inhibit the N-myristoyl transferase of *C. albicans* and the *S. mutans* antigen III and may thus contribute to the eradication of oral infections.

The cytotoxic effect and dose-dependent cell toxic effect of PGLEO on the cells of KB was confirmed by MTT assay. The cytotoxic effect of PGLEO was observed in the cells of KB, suggesting that volatile compounds have increased cytotoxicity in KB oral cancer cells. The IC_50_ value of doxorubicin (0.18 ± 0.13 µg/mL) has been reported for KB cells in a previous study [55]; the IC_50_ value of PGLEO is very high compared to standard doxorubicin. Our results are in agreement with previous reports [56]. Working with Thai *Psidium guajava*, Manosroi et al. (2006) showed the significant cytotoxic effect of PGLEO at different doses and its antiproliferative effect against KB cells for 24 h. In contrast, the suppressive effect was greater than vincristine and essential basil oil [24]. In the present work, in vitro and in silico studies were carried out on oral microorganisms (*C. albicans* and *S. mutans*), and the anticancer effect of PGLEO on human mouth epidermal carcinoma KB cell line was investigated. The outcome of the study shows the role of PGLEO in overcoming oral problems such as dental caries and oral cancer. PGLEO may be a useful source in future drug development, especially for managing oral conditions.

## 4. Materials and Methods

### 4.1. Chemicals

The human oral adenocarcinoma cell line KB was obtained from the National Center for Cell Science (NCCS, Pune). Dulbecco’s modified Eagle’s high-glucose medium (DMEM-HG; Cat No: AL007), fetal bovine serum (FBS), MTT reagent, and Dulbecco’s phosphate-buffered saline (D-PBS) were purchased from HiMedia Laboratories (Mumbai, India). Dimethyl sulfoxide (DMSO) and doxorubicin were purchased from Sigma-Aldrich (Mumbai, India). All media used for the antimicrobial studies were purchased from HiMedia Laboratories (Mumbai, India). Analytical-grade chemicals were purchased from HiMedia, Sigma Aldrich, and Merck (Mumbai, India), and 96-well plates for cell culture were purchased from Corning (Kennebunk, ME, USA).

### 4.2. Extraction of Essential Oils

Guava (*P. guajava*) leaves were collected from Integral College (Lucknow, Uttar Pradesh, India) in November 2021. The sample was authenticated (IU/PHAR/HRBD 22/03) and deposited at the College of Pharmacy, Integral College. The leaves were dried and powdered. Then, the essential oil was extracted using a Clevenger-type apparatus by the hydrodistillation method as described in a previous study [57]. The extracted essential oil was dried with anhydrous sodium carbonate; it was weighed and stored in an airtight amber vial at −20 °C (under refrigeration) until use.

### 4.3. Characterization of PGLEO Using Gas Chromatography–Mass Spectrometry

Chemical characterization was performed by gas chromatography and mass spectrometry. A gas chromatograph–mass spectrometer model QP2010 (Shimadzu, Tokyo, Japan) equipped with an autosampler and autoinjector (AOC-20si) was used for this study. The procedures for injection, separation, and identification of compounds, as well as the temperature of the column oven, were based on previous reports with some modifications [58,59]. The essential oil was dissolved in hexane (10 mg/mL), and the system was operated at 70 eV. This solution (1 µL) was injected in split mode (1:10 ratio) onto a Rtx-5MS column (Shimadzu, Japan) with the following dimensions: stationary phase film thickness of 0.25 µm, length of 30 m, and diameter of 0.25 mm. The injection temperature was set at 260 °C. The oven temperature was started at 50 °C for 2 min after injection and then increased at 5 °C/min to 180 °C for 1 min, followed by an increase at 15 °C/min to 280 °C, where the column was held for 15 min. The helium gas flow rate with a column head pressure of 69 kPa was set to 1.21 mL/min. Mass spectra were obtained in the range of 40 to 650 m/z. Essential oil components were identified based on a search (National Institute of Standards and Technology, NIST 14), the calculation of retention indices relative to homologous series of n-alkane (C7-C30), and a comparison of their mass spectra libraries with data from the mass spectra in the literature [60,61].

### 4.4. Effects of PGLEO on Oral Pathogen

The antimicrobial activity of PGLEO was tested against *Streptococcus mutans* (MTCC 389) and the fungus *C. albicans* (MTCC 9215). The antimicrobial activity of PGLEO was tested using the well diffusion method [62] with some modifications, and each experiment was evaluated in triplicate. Nutrient agar and Sabouraud dextrose agar medium were used for the antimicrobial assay. *S. mutans* cultures were separately inoculated into the nutrient broth and cultured at 37 °C for 18 h, while *C. albicans* subcultures were inoculated into Sabouraud dextrose broth and cultured at 37 °C for 48 h. The suspensions of all test organisms were diluted separately with a phosphate buffer (pH 7.4) to obtain 1 × 10^8^ colony-forming units/mL of microbial suspension.

The inoculum (50 µL) from the broth was poured onto a fresh, sterile, solidified agar medium plate using a micropipette. Four 6 mm wells were drilled into the inoculated medium using a sterile cork borer. In addition, 2% dimethyl sulfoxide (DMSO) was used for sample preparation. Each well was filled with a 50 µL sample at different concentrations (0.25%, 5%, 7.5%, and 10%). Amoxicillin and fluconazole (0.001% in 2% DMSO) were used as positive controls for antibacterial and antifungal analyses, respectively, while 2% DMSO was used as a negative control. Plates were labelled and samples were placed in the wells of the plates and incubated at 37 °C for 24 and 48 h for bacteria and fungi, respectively.

The diameter of the inhibition (inhibition zone) around the wells was measured, and the mean ± standard deviation was calculated for each of the three assays. Different concentrations (0.0125, 0.025, 0.05, 0.1, and 0.2%) of essential oil were prepared in 2% DMSO and used to determine the minimum inhibitory concentration (MIC) using the broth dilution method [63]. Next, 100 µL of inoculum was added to each diluted tube, and the control tubes (no bacterial inoculation) were evaluated simultaneously. All tubes with antibacterial activity were incubated at 37 °C for 24 h, and the tubes with antifungal activity were incubated at 37 °C for 48 h; the lowest concentration resulted in no visible growth.

To investigate the bactericidal and fungicidal activity of PGLEO, the time-kill assay of Foudah et al. was performed [57]. *S. mutans* cultures were incubated on nutrient broth agar, while *C. albicans* was inoculated into Sabouraud dextrose broth and cultured at 37 °C for 8 h. The suspension was centrifuged and resuspended in saline at 10^6^ CFU/mL. The suspensions of *S. mutans* and *C. albicans* were treated with broth medium containing different concentrations of PGLEO according to the MIC, mixed, and cultured at 37 °C. Samples were removed from the culture at selected time intervals (0, 4, 8, 16, and 24 h), diluted with saline, and recultured in a broth medium. After incubation of the plates at 37 °C for 24 h, the colony-forming unit/mL (CFU/mL) was calculated and a graph of CFU/mL versus time was plotted to calculate the time-kill assay.

### 4.5. Molecular Coupling Effects of Major Compounds on the Receptors for S. mutans and C. albicans

In this study, the two primary compounds, D-limonene and β-caryophyllene, were selected for molecular coupling to evaluate their effects on *S. mutans* and *C. albicans*. The AutoDock (ADT) tool created the file from the conventional RCSB PDB files [64]. For the molecular linkage study, 1JMM: region V of *S. mutans* antigen III was selected for the inhibitory effect of *S. mutans* and 1IYL: *C. albicans* N-myristoyl transferase with nonpeptide inhibitor was selected for the inhibitory effect of *C. albicans* [65]. To prepare the 3D input files, only the protein part of the PDB structures was selected by removing unwanted atoms, ions, and molecules [66]. All target proteins were energetically minimized by applying the CHARMm force field [67,68]. The 2D structures of selected ligands, including β-caryophyllene (CID: 5281515) and D-limonene (CID: 440917), in standard data format (SDF) were retrieved from the PubChem database [69]. SDF-2D was converted to PDB-3D using the BIOVIA discovery studio visualizer [70], and all ligands were energy minimized following the same protocol as the receptor molecules [68].

The molecular interactions of the ligands (β-caryophyllene and D-limonene) and the 3D structure of the protein molecules were performed using ADT to determine their potential binding affinities. The receptors and ligands, a grid parameter file, and docking parameter PDF files were created to perform the docking experiments. The grid frame around the protein molecule was drawn with variable grid points on the x, y, and z axes and a maximum distance (1.00) between two consecutive grids to provide sufficient space for ligand movement. Ten runs were performed for each ligand as standard. The minimum binding free energy and inhibition constant (Ki) was considered selective parameters to choose one of the best poses of the ligands bound to the binding cleft of the receptor molecules [71].

### 4.6. Cytotoxicity Assay

Human mouth epidermal carcinoma (KB) cells were maintained in high glucose medium supplemented with 10% FBS (fetal bovine serum) and 1% antibiotic–antifungal solution in an atmosphere of 5% CO_2_ and 18–20% O_2_ at 37 °C in a CO_2_ incubator (Healforce, Shanghai, China) and subcultured every two days. The human oral adenocarcinoma cell line was established according to a previous experiment [69] with some modifications. First, 200 μL cell suspension was seeded into a 96-well plate (2 × 10^4^ cells/well), and the cells were allowed to grow for approximately 24 h. Then, appropriate concentrations of the test samples were added and incubated for 24 h at 37 °C in a 5% CO_2_ atmosphere. The plates were removed from the incubator, the spent medium was removed, and 3-(4,5-dimethylthiazol-2-yl)-2,5-diphenyl-2H-tetrazolium bromide (MTT reagent) was added at a final concentration of 0.05% of total volume. The plate was wrapped with aluminum foil and incubated for 3 h. The MTT reagent was then removed, 100 μL DMSO was carefully added, and the absorbance of the plates was read using an enzyme-linked immunosorbent assay (ELISA) plate reader (Biorad-PW41, Hercules, CA USA) at 570 nm. Percentage cell viability was calculated using the formula below, and IC_50_ was determined using a linear regression equation Y = Mx + C, where Y = 50 and M and C values were derived from the viability diagram.

% cell viability= [Abs of treated cells/Abs of untreated cells] × 100(1)

Morphological changes and cell death of apoptotic cells were studied by fluorescence microscopy. At different concentrations (0.00125%–0.02%), cells were visualized after treatment of KB with PGLEO using fluorescence microscopy (Leica Microsystems GmbH) at a magnification of 100×. Cells with condensed and fragmented nuclei were considered apoptotic cells.

### 4.7. Statistical Analysis

Data were analyzed using GraphPad InStat (La Jolla, CA, USA) software and are presented as mean ± standard deviation. The essential oil concentration that provides 50% inhibition (IC_50_ value) was calculated using the graph by plotting the inhibition percentage versus the essential oil concentration.

## 5. Conclusions

This study investigated the effects of PGLEO on common oral pathogens and oral cancer. The oil showed solid antibacterial properties against *S. mutans* and *C. albicans*. Molecular docking studies of the primary compounds (limonene and β-caryophyllene) of PGLEO with the proteins 1JMM and 1IYL revealed theoretical inhibitory effects on the target proteins of *S. mutans* and *C. albicans*, consistent with the present antimicrobial activities, as indicated by their significant protein–ligand interaction energy. In addition to antibacterial activity, PGLEO also exhibited oral anticancer effects. Therefore, the present results support the potential use of PGLEO as an oral hygiene, anticariogenic, and anticarcinogenic agent.

## Figures and Tables

**Table 1:** Volatile composition of the PGLEO by GC–MS analysis.

Volatile Composition	Types	RI (Lit)	RI (Obs)	Area %
α-Pinene	MH	949	948	0.71
Myrcene	MH	963	958	0.57
β-Ocimene	MH	986	976	0.58
D-Limonene	MH	1022	1018	38.01
Menthol	OM	1173	1164	0.20
Linalyl acetate	OM	1270	1272	0.30
α-Cubebene	SH	1347	1344	0.98
Copaene	SH	1377	1371	6.25
α-cedrene	SH	1415.1	1403	1.62
β-Caryophyllene	SH	1419	1414	27.98
α-Caryophyllene	SH	1446	1440	4.21
Cadina-3,5-diene	SH	1454	1448	0.23
γ-Muurolene	SH	1475	1465	1.19
β-Selinene	SH	1492	1490	0.19
α-Bisabolene	SH	1511	1500	1.29
Cubinene	SH	1515	1510	4.80
Cadina-1,3,5-triene	SH	1542	1537	0.80
Ledol	OS	1540	1530	0.96
Nerolidol	OS	1565	1564	4.56
Caryophyllene oxide	OS	1580	1570	2.17
α-Muurolol	OS	1611	1580	0.42
Cholestadiene	D	-	2390	0.22
2-Hexadecen-1-ol, 3,7,11,15-tetramethyl	NT	2114	2114	0.69
13-Hexyloxacyclotridec-10-en-2-one	NT	2325	2325	1.08
Monoterpene hydrocarbon (MH)			39.87	
Oxygenated monoterpenes (OM)			0.5	
Sesquiterpenes hydrocarbon (SH)			49.54	
Oxygenated sesquiterpenes (OS)			8.11	
Diterpenes (D)			0.22	
Nonterpene (NT)			1.77	
Total identification (34 components)			100.01%	

RI (Obs): calculated retention index; RI (Lit) = retention indices according to Adams (Adams, 2007).

**Table 2:** Antimicrobial activity of *Psidium guajava*.

Concentration (%)	Zone of Inhibition (mm, Mean ± SD)	
	*S. mutans*	*C. albicans*
0.25	Less than 6mm	Less than 6mm
0.5	9.5 ± 0.7	10 ± 0.4
7.5	15.5 ± 0.7	16.5 ± 0.7
10	18.5 ± 0.7	21.5 ± 0.5
Minimum inhibitory concentration (MIC)	0.05–0.1%	0.05–0.1%

Values were expressed as mean ± SD (standard deviation) calculation (n = 3).

**Table 3:** Binding energy (ΔG; kcal/mol) and inhibition constant (Ki; µM) for limonene (LIM) and caryophyllene (CRP) with target proteins.

Targets (PDB)	ΔG		Ki		Residues of Amino Acid	
	LIM	CRP	LIM	CRP	D-Limonene (LIM)	β-Caryophyllene (CRP)
**1JMM**	−7.42	−7.39	785.97	876.32	PHE A:192, ILE A:345, ALA A:319	ILE A:345, ALA A:319
**1IYL**	−8.03	−8.21	752.10	675.29	TYR A:287, TYR A:158, PHE A:50, PHE A:272, PHE A:48, PHE A:173	PHE A:173, HIS A:160, TRP A:165

## Data Availability

Not applicable.
